# Complete Genome Sequence of a Novel Reassortant Avian Influenza H9N9 Virus Isolated from Chicken in Eastern China

**DOI:** 10.1128/genomeA.00932-14

**Published:** 2014-09-25

**Authors:** Xiling Guo, Xian Qi, Lunbiao Cui, Yiyue Ge, Huiyan Yu, Xiaojuan Zhu, Yin Chen, Zhiyang Shi, Minghao Zhou

**Affiliations:** Key Lab of Enteric Pathogenic Microbiology (Ministry of Health), Institute of Pathogenic Microbiology, Jiangsu Provincial Center for Disease Prevention and Control, Nanjing, China

## Abstract

The genome sequence of the strain A/chicken/Changzhou/C08/2013 (H9N9) shows that the hemagglutinin (HA) genes of this strain are closely related to those of the strain A/chicken/Shanghai/1107/2013 (H9N2) and share 99.2% nucleotide homology, while the other seven genes had the greatest sequence identities with the novel H7N9 virus. We speculate that this strain may be a novel natural reassortant avian influenza virus (AIV).

## GENOME ANNOUNCEMENT

Since March 2013, outbreak of a novel reassortant avian influenza virus (AIV), H7N9, has caused severe public health problems in China (1). The hemagglutinin (HA) and neuraminidase (NA) genes of this virus are believed to originate from Eurasian H7 and N9 AIV subtypes, and the six internal genes are from the H9N2 AIV subtypes (2). H7N9 virus is still undergoing dynamic internal gene reassortment with the local avian H9N2 viruses (3, 4). Scientific communities should pay more attention to the reassortant events of this virus, which may lead to the emergence of new reassortant viruses.

In December 2013, an H9N9 AIV strain, A/chicken/Changzhou/C08/2013 (H9N9) (C08), was isolated from chicken in Jiangsu, China. The eight genes were amplified by reverse transcription-PCR (RT-PCR) using a set of universal primers (5). The PCR products were sequenced by MiSeq (Illumina). Sequences were assembled by CLC Genomics workbench (CLC Bio).

The full lengths of the PB2, PB1, PA, HA, NP, NA, M, and NS genes were 2,341, 2,341, 2,233, 1,742, 1,565, 1,444, 1,027, and 890 nucleotides, respectively. The amino acid sequence of the cleavage site in the HA protein was VPSRSSR↓GLF, with the characteristics of low-pathogenicity AIV. The amino acid residue at the receptor binding site 226 (H3 numbering) in the HA protein was leucine (L), which is characteristic of the mammalian influenza virus. However, the amino acid residue at another receptor binding site 228 (H3 numbering) in the HA protein was glycine (G), which preferentially binds to the AIV receptor. The amino acids at residues 591, 627, and 701 in the PB2 protein were glutamine (Q), glutamic acid (E), and aspartic acid (D), respectively, a characteristic of avian replication preference. There are 13 amino acid deletions in the NS1 genes and 5 amino acid deletions at the NA stalk region, as observed in novel H7N9 viruses (3).

Sequence analysis showed that the nucleotide sequences of the HA gene of the C08 strain are closely related to those of the A/chicken/Shanghai/1107/2013 (H9N2) strain and share 99.2% nucleotide homology, while the other seven genes have the greatest sequence identities (over 99%) with the novel H7N9 virus. Therefore, we speculate that the C08 strain may be a novel natural reassortant virus, with its HA gene from H9N2 and the other seven genes from H7N9 AIVs.

In conclusion, H7N9 AIVs are undergoing reassortment with other AIV epidemic strains in birds, such as the H9N2 virus, which highlights the importance of surveillance of the evolution of the H7N9 virus.

### Nucleotide sequence accession numbers.

The genome sequences of A/chicken/Changzhou/C08/2013 (H9N9) have been deposited in GenBank under the accession numbers KJ938647 to KJ938654.
